# Establishing a filarial clinical research platform in a resource-limited setting: Lessons and experiences from Tanzania

**DOI:** 10.1371/journal.pntd.0013983

**Published:** 2026-02-09

**Authors:** Ndekya M. Oriyo, Michael A. Munga, Abdallah Ngenya, John Ogondiek, Wilfred Mandara, Winfrida Shirima, Jonathan Akyoo, Ruth Laizer, Mathias Kamugisha, Hanifa Kapera, Rafikieli Ngwatu, Gracia Sanga, Jacklina Mhidze, Antelmo Haule, Anja Feichtner, Haile Mposheleye, Farida A. Mwanga, Hatima A. Chinguile, Alexander J. Makalla, Peter Mabenga, Max Demetrius, Maureen M. Mosoba, Dennis Moshi, Yusuph Mgaya, Linda Batsa Debrah, Inge Kroidl, Ute Klarmann-Schulz, Samuel Wanji, Alexander Yaw Debrah, Achim Hoerauf, Akili Kalinga, Upendo Mwingira

**Affiliations:** 1 National Institute for Medical Research, Dar es Salaam, Tanzania; 2 Kilimanjaro Clinical Research Institute, Moshi, Tanzania; 3 National Institute for Medical Research (NIMR), Mbeya Medical Research Centre, Mbeya, Tanzania; 4 Division of Infectious Diseases and Tropical Medicine, University Hospital Munich, Ludwig-Maximilians-University (LMU), Munich, Germany; 5 Sokoine Regional Referral Hospital, Lindi, Tanzania; 6 Mbanja Dispensary, Lindi, Tanzania; 7 Kumasi Centre for Collaborative Research in Tropical Medicine (KCCR), Kwame Nkrumah University of Science and Technology (KNUST), Kumasi, Ghana; 8 Department of Clinical Microbiology, School of Medicine and Dentistry, Kwame Nkrumah University of Science and Technology (KNUST), Kumasi, Ghana; 9 German Center for Infection Research (DZIF), Neglected Tropical Diseases, Partner Site, Munich, Germany; 10 Institute for Medical Microbiology, Immunology and Parasitology (IMMIP), University Hospital Bonn, Bonn, Germany; 11 German Center for Infection Research (DZIF), Bonn-Cologne, Germany; 12 Institute for Medical Biometry, Informatics and Epidemiology (IMBIE), University Hospital Bonn, Bonn, Germany; 13 Department of Microbiology and Parasitology, University of Buea, Buea, Cameroon; 14 Faculty of Allied Health Sciences, Kwame Nkrumah University of Science and Technology (KNUST), Kumasi, Ghana; 15 German Center for Infection Research (DZIF), Neglected Tropical Diseases, Partner Site, Bonn-Cologne, Bonn, Germany; 16 German-West African Center for Global Health and Pandemic Prevention (G-WAC), Partner Site, Bonn, Germany; 17 RTI International, Washington, DC, United States of America; Bayer AG, SWITZERLAND

## Abstract

This paper examines the establishment and lessons learned from the LeDoxy Trial, a multinational, double-blind, randomized, placebo-controlled study that evaluated doxycycline (200 mg/d and 100 mg/d) over six weeks for the treatment of filarial lymphedema in Tanzania’s Lindi and Pwani regions. Conducted from 2018 to 2021 across four sites, the trial enrolled 420 participants aged 14–65 years, addressing a critical gap in lymphatic filariasis (LF) research in geographically remote and historically under-researched areas. The trial faced significant challenges, including limited pre-existing research infrastructure, community research fatigue, and disruptions from the COVID-19 pandemic, which delayed regulatory approvals and supply chains. To overcome these, the TAKeOFF Consortium with Tanzania’s National Institute for Medical Research (NIMR) as local sponsor led a 12-month site preparation effort, upgrading Lindi’s laboratory from a 2-star to a 4-star rating through infrastructure enhancements (e.g., centrifuges, freezers) and staff training in Good Clinical Laboratory Practice (GCLP). Community health workers (CHWs) leveraging established community-directed approaches from NTD control programs played a pivotal role, achieving a 92% retention rate through targeted recruitment, door-to-door engagement, and follow-up support, including the distribution of hygiene kits. The trial trained 93 healthcare professionals in LF management and clinical research, fostering a research culture despite initial resistance. Additional strategies included remote monitoring to adapt to travel restrictions and stakeholder collaboration to address cultural misconceptions, such as stigma around LF. These efforts not only ensured trial success but also created foundational capacity that has begun to support post-trial research activities, with ongoing evaluation needed to assess long-term sustainability. This paper provides comprehensive, integrated documentation of the infrastructure development process from laboratory accreditation and regulatory navigation to community engagement and sustainability planning, offering actionable guidance for conducting high-quality NTD trials in resource-limited settings. The lessons emphasizing local sponsorship, adaptive protocols, and community trust demonstrate how strategic investments in research infrastructure, local capacity-building, and stakeholder engagement can strengthen clinical research ecosystems in LMICs, advancing health systems, local ownership, and global health equity. This paper focuses exclusively on the operational and implementation aspects of establishing the trial infrastructure.

## Author summary

Clinical trials are essential for developing better treatments for neglected tropical diseases, yet they are rarely conducted in remote and underserved regions where disease burden is often highest. This paper describes how a clinical research platform was successfully established in Lindi, a rural region of southern Tanzania with no prior experience hosting clinical trials, as part of the LeDoxy Trial for lymphatic filariasis. Rather than reporting clinical outcomes, this article focuses on the practical challenges encountered and the solutions developed while preparing and implementing the trial. Key challenges included limited laboratory infrastructure, shortages of trained research personnel, complex regulatory requirements, community research fatigue, and disruptions caused by the COVID-19 pandemic. Through a locally led approach, Tanzania’s National Institute for Medical Research assumed the role of trial sponsor and worked with national and international partners to upgrade laboratory facilities, train healthcare and research staff, strengthen regulatory oversight, and engage communities through trusted community health workers. These efforts resulted in high participant retention and the establishment of research capacity that extended beyond the trial period. By documenting these experiences, this paper provides practical guidance for researchers, policymakers, and sponsors seeking to conduct high-quality clinical trials in resource-limited settings, demonstrating how strategic investments in local leadership, infrastructure, and community engagement can strengthen health systems and promote equitable global health research.

## Introduction

Clinical trials are fundamental for advancing medical knowledge, evaluating new treatments, and verifying the efficacy and safety of investigational products across diverse populations [1–3]. Beyond their immediate scientific contributions, clinical trials also play a crucial role in strengthening health services and research infrastructure within countries. However, initiating and executing clinical trials, particularly in resource-limited settings, is fraught with challenges that demand meticulous planning and innovative solutions.

Conducting interventional clinical trials involves a complex and resource-intensive process. From site preparation and initiation to participant recruitment, follow-up, and study close-out, each phase requires rigorous oversight and adherence to both international standards and local regulations. In regions with limited infrastructure, expertise, and experience, these challenges are amplified, making the conduct of such trials particularly daunting.

In sub-Saharan Africa, including Tanzania, significant progress has been made in the realm of clinical research. While some disease-specific research activities have been concentrated in selected locations, many geographically remote and high-burden areas remain under-researched due to limited infrastructure, logistical constraints, and insufficient research capacity [4,5]. In such settings, research exposure is often sporadic rather than sustained, resulting in both gaps in evidence generation and limited opportunities for local capacity development [6,7]. Conversely, regions with high disease burdens frequently lack the necessary research infrastructure and expertise, making them less attractive to researchers and funders despite clear public health need.

Unlike previous research initiatives in Tanzania, which focused on urban or semi-urban areas, this trial represents one of the first efforts to establish a clinical research site in a remote, underserved region, offering unique insights into resource-limited settings. This approach not only addresses an existing gap in clinical research but also provides valuable lessons for scaling research capabilities in similar contexts globally. Additionally, most previous trials in Tanzania have been led by international sponsors with limited local ownership. This study is distinct in that it was led by Tanzania’s National Institute for Medical Research (NIMR) which is part of the Tackling the Obstacles to Fight Filarial Infections and Podoconiosis (TAKeOFF) Consortium, a collaborative network of international experts from five research institutions, established to address filarial infections and podoconiosis by developing innovative treatment and morbidity management strategies through a dedicated filarial clinical trial and research platform. This model represents an important step toward strengthening locally led trial sponsorship and reducing dependence on external entities for trial leadership and oversight in LMICs.

Globally, lymphatic filariasis (LF) remains a major neglected tropical disease, with hundreds of millions of people still requiring preventive chemotherapy despite substantial progress toward elimination in several endemic countries [8]. While mass drug administration (MDA) has reduced transmission in many settings, a significant proportion of affected individuals continue to experience chronic morbidity, including lymphoedema and hydrocele, underscoring the need for effective morbidity management strategies.

Lymphatic filariasis remains a public health concern in Tanzania, with lymphoedema persisting despite mass drug administration (MDA). In Lindi District, the prevalence of lymphoedema was 7.8%, with the highest incidence among individuals aged 53–68 years. Most cases were early-stage (78.4%), while 21.7% had advanced lymphoedema. Individuals with poor hygiene were 7.38 times more likely to develop advanced lymphoedema (AOR = 7.38, p = 0.04) [9]. This burden, coupled with the absence of prior clinical research infrastructure, informed the selection of Lindi as a primary study site for the LeDoxy Trial.

Pwani Region was selected as a complementary study site due to its documented lymphatic filariasis endemicity, established participation in national mass drug administration programs, and relatively greater accessibility compared to Lindi. Inclusion of Pwani was intended to provide an operational contrast to Lindi, enabling the trial to examine implementation experiences across two epidemiologically similar but infrastructurally and logistically distinct settings. This approach strengthened the assessment of feasibility, adaptability, and transferability of site preparation and trial management strategies while maintaining focus on high-burden regions.

The LeDoxy Trial, part of the TAKeOFF Consortium, evaluated Doxycycline (200 mg/d vs. 100 mg/d) for filarial lymphedema in a multinational, double-blind, randomized, placebo-controlled study in Tanzania’s Lindi and Pwani regions. The trial’s clinical outcomes, including efficacy in arresting or reversing lymphedema progression, are reported in Ngenya et al [10]. This manuscript focuses exclusively on the operational and implementation experiences associated with establishing a clinical research platform in high-burden, under-researched settings. By centering on Lindi, a region with no prior experience hosting clinical trials, and complementing it with Pwani, the study offers unique insights into both foundational capacity building and adaptive trial implementation within the Tanzanian context.

This paper aims to illuminate the experiences and lessons learned from setting up and managing the LeDoxy Trial in such a resource-constrained environment. By sharing these insights, we aim to offer guidance to other researchers undertaking similar endeavors and provide a deeper understanding of the complexities involved in conducting clinical trials in resource-constrained regions. The lessons from the LeDoxy Trial offer critical insights for designing and implementing clinical trials in underserved regions, demonstrating how strategic investments in research infrastructure, local capacity-building, and stakeholder engagement can strengthen clinical research ecosystems in LMICs. This, in turn, paves the way for more locally led trials and sustainable health system improvements.

## Methods

### Ethics statement

Ethical approval for the parent LeDoxy Trial was obtained from the National Health Research Ethics Committee of the Tanzania Medical Research Coordinating Committee, with certificate number NIMR/HQ/R.8a/Vol. IX/2693. Clinical trial approval was granted by the Tanzania Medicines and Medical Devices Authority, under certificate number TFDA0017/CTR/0020/3.

As the study included adolescent participants aged 14–17 years, formal written informed consent was obtained from all participants aged 18 years and above. For participants under 18 years of age, written informed assent was obtained from the adolescent, alongside written informed consent from a parent or legal guardian.

For participants who could not read or write, the information sheet and consent form were read aloud in the presence of an impartial witness. Consent was documented using the participant’s thumbprint, countersigned by the witness.

### Study design and paper classification

This paper is a structured implementation and experiential analysis documenting the processes, challenges, and lessons learned from establishing and operationalizing the LeDoxy clinical trial platform in a resource-limited setting in Tanzania. It does not report clinical efficacy or safety outcomes of the LeDoxy Trial, which are published elsewhere. Rather, it focuses on the operational, infrastructural, regulatory, and human resource dimensions involved in setting up and managing a complex interventional clinical trial in a previously under-researched region.

Accordingly, this work does not follow a hypothesis-driven or comparative study design. Instead, it adopts a retrospective, descriptive analytical approach grounded in systematic documentation, triangulation of implementation records, and reflective synthesis of experiences generated during trial preparation, execution, and close-out.

### Relationship to the parent LeDoxy Trial

The LeDoxy Trial was a multinational, double-blind, randomized, placebo-controlled clinical trial evaluating doxycycline for the management of filarial lymphedema, conducted between 2018 and 2021 in Tanzania’s Lindi and Pwani regions. While the parent trial generated clinical and laboratory data subject to formal statistical analysis, the present paper draws exclusively on non-clinical implementation data and operational experiences associated with establishing and running the trial platform. No participant-level clinical outcome data were analyzed for this manuscript.

### Data sources

Multiple sources of information generated throughout the lifecycle of the LeDoxy Trial were systematically reviewed and synthesized to inform this analysis. These sources included site readiness and feasibility assessment reports; laboratory accreditation and quality improvement documentation, including records from the Stepwise Laboratory Improvement Process Towards Accreditation (SLIPTA); and training-related materials such as training plans, curricula, attendance registers, and post-training evaluation reports. Regulatory and ethics correspondence with national oversight authorities was also reviewed, alongside monitoring and supervision reports from both remote and on-site visits. Additional sources comprised trial management records documenting staffing arrangements, logistics, and infrastructure upgrades, as well as community engagement records, including summaries of stakeholder meetings and reports from community health worker (CHW) activities. Oversight documentation maintained by the National Institute for Medical Research (NIMR) in its role as trial sponsor was also included. All materials were generated as part of routine trial governance, quality assurance, and regulatory compliance processes and did not involve the collection of new primary data for the purposes of this manuscript.

### Perspective and analytical lens

The analysis reflects the collective perspectives of the trial sponsor, site leadership, and core implementation team. Authors involved in trial sponsorship, coordination, laboratory oversight, regulatory management, and field implementation contributed to the synthesis of lessons learned. These perspectives reflect operational roles held during trial preparation, implementation, monitoring, and close-out. To reduce reliance on individual recollection, experiential insights derived from documented implementation processes were cross-checked against contemporaneous written records wherever available. The analytical lens was informed by established principles of clinical trial implementation, health systems strengthening, and research capacity building in low- and middle-income country contexts.

### Analytical process

A structured thematic synthesis approach was employed to analyze the implementation experiences documented during the LeDoxy Trial, drawing exclusively on implementation documentation rather than primary interview or focus group data. The analysis began with an initial mapping of key implementation domains aligned with the major phases of the trial, including site preparation, participant recruitment, trial execution, monitoring, and sustainability planning. Recurrent challenges, enabling factors, and mitigation strategies were then systematically identified from the reviewed documentation. These observations were subsequently organized into thematic domains encompassing governance and sponsorship, human resource management, laboratory and infrastructure development, regulatory processes, community engagement, participant recruitment and retention, and adaptive responses to external disruptions, including the COVID-19 pandemic. The thematic framework was iteratively refined through internal discussion among the authors to ensure coherence, internal consistency, and alignment with the available documentary evidence. The final thematic structure informed the organization and presentation of the Results and Implementation section.

### Ensuring analytical rigor and trustworthiness

Several measures were employed to enhance the credibility and trustworthiness of the analysis. Triangulation was undertaken across multiple documentary sources and stakeholder perspectives to ensure consistency and completeness of the observations reported. Wherever possible, contemporaneous trial records were prioritized over retrospective recollection to minimize recall bias. Key claims and interpretations were cross-validated against monitoring and supervision reports, training documentation, and laboratory accreditation records. Throughout the analysis, a clear distinction was maintained between empirically observed implementation outcomes and the authors’ interpretive reflections on those experiences. In addition, the non-comparative and descriptive nature of the analysis was explicitly acknowledged, ensuring that findings are presented as observational and experience-based rather than inferential. These measures were selected to enhance rigor within a descriptive, non-comparative implementation analysis rather than to support inferential or hypothesis-driven claims.

## Results and implementation

### Recruiting clinical trial research teams

In LMICs, clinical trial staff often face the challenge of balancing dual responsibilities providing routine healthcare services and managing research activities. This dual focus can create tensions that may impact either the quality of patient care or the integrity of the clinical trial [11]. To mitigate this risk, it was essential to negotiate adequate time allocation and resource management with healthcare facility authorities prior to the commencement of the trial. This step was crucial to ensure that research activities did not interfere with ongoing patient care.

A key component of recruitment was identifying and training personnel capable of assuming leadership roles, as well as cultivating a strong research culture at the trial sites. To achieve this, we established clear selection criteria based on specific qualifications and experience levels, as outlined in NIMR staff regulations, ensuring alignment with the expertise required for each role. This approach not only ensured that the research team comprised individuals with the necessary skills and commitment to conduct the trial effectively, but also guaranteed that project staff remained eligible for future research and employment opportunities at NIMR after the study’s conclusion.

**Fig 1:**
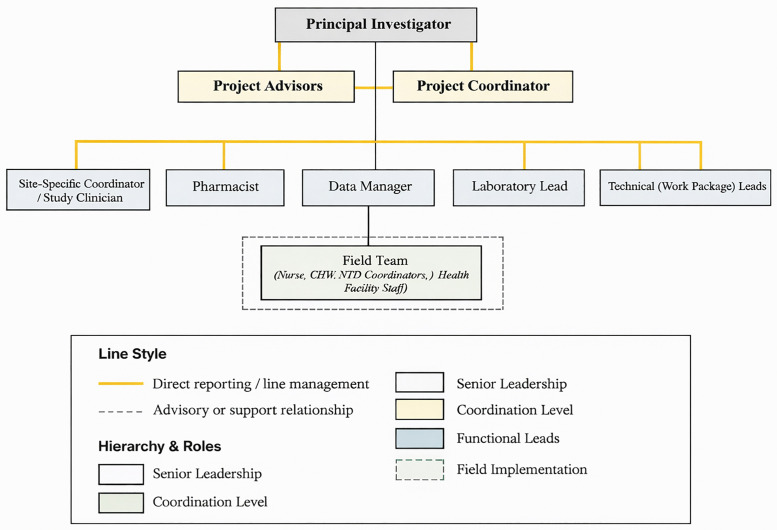
Organizational structure of the LeDoxy Trial showing formal reporting and supervisory relationships across leadership, coordination, functional teams, and field implementation levels.

The recruitment strategy focused on including both researchers and study team members from the local health facilities and communities where the trial would be conducted as shown in Fig 1. By setting clear criteria for staff qualifications, we aimed to build a team that not only met the technical requirements of the trial but also integrated well with the local healthcare system. This approach not only facilitated the recruitment of competent staff but also garnered the support of local authorities and healthcare teams, fostering a sense of ownership and commitment to the trial’s success.

Moreover, comprehensive training programs combining theoretical and practical components were implemented to equip the recruited staff with the necessary skills and knowledge. The theoretical component covered fundamental principles of clinical research, Good Clinical Practice (GCP) guidelines, and the specific protocol requirements of the LeDoxy trial. Practical training included hands-on sessions in patient assessment, data collection procedures, laboratory techniques specific to filarial diagnostics, and adverse event monitoring. This training encompassed not only the technical aspects of clinical research but also the cultural and contextual nuances of working in a resource-limited setting. Emphasizing the importance of building a scientific and research-oriented culture, the training aimed to align the staff’s efforts with the trial’s objectives while maintaining a high standard of patient care.

Therefore, effective recruitment and training of clinical trial staff in LMICs involves careful planning and negotiation to balance research and service responsibilities. By setting minimum requirements for qualifications and investing in comprehensive training, we were able to build a capable and committed team, essential for the successful implementation of the trial.

### Study site selection and preparedness

Selecting and preparing study sites for clinical trials in resource-limited settings involves a series of strategic steps to ensure compliance with international standards and local requirements. A critical component of this process is the development of a standard clinical trial checklist, which outlines the essential criteria and operational requirements for trial sites. This checklist serves as a guide for identifying and preparing facilities that can effectively support the trial’s needs.

In our study, significant challenges arose in locating laboratory facilities that were enrolled in an accreditation program, a crucial requirement for ensuring the quality and reliability of trial data. Since 2015, government health facilities in Tanzania have participated in the WHO AFRO program, which employs the Stepwise Laboratory Improvement Process Towards Accreditation (SLIPTA) to enhance laboratory quality across the African region. This program uses a 5-star rating system to evaluate facilities. Higher star ratings reflect improved adherence to good laboratory practices, biosafety, documentation, and quality management systems [12].

At the start of the TAKeOFF project, the Sokoine Regional Referral Hospital laboratory in Lindi had an SLIPTA rating of 2 stars. Through a deliberate capacity-building strategy, the project facilitated its upgrade to 4 stars and eventual full accreditation by the Southern African Development Community Accreditation Services (SADCAS). This improvement was achieved through a combination of targeted staff training on GCLP, infrastructure upgrades that included the provision of centrifuges, deep freezers (-20°C and -80°C), and liquid nitrogen tanks, as well as the establishment of internal quality management systems aligned with ISO standards. Regular supportive supervision and mentorship were provided to reinforce adherence to standardized operating procedures and continuous quality improvement practices. These interventions significantly enhanced the laboratory’s readiness to support the trial.

To meet the GCP standards required for the trial, it was necessary to select facilities with a star rating of at least 3 under the WHO AFRO SLIPTA accreditation program. The study team identified Sokoine Hospital, a regional referral hospital in Lindi, as the primary site for clinical, laboratory, and pharmacy functions. The Lindi region was chosen due to its high burden of lymphatic filariasis, lack of prior clinical research infrastructure, and its strategic importance for expanding health services in southern Tanzania. However, given that the Lindi region had limited options for qualified health facilities, it was essential to establish a backup plan. Consequently, St. Benedict Ndanda Hospital, located in the neighboring Mtwara region, approximately 104 kilometers away, was designated as the secondary laboratory site to ensure continuity and mitigate potential disruptions at the primary location. The selection was based on the fact that the hospital was equipped with a backup generator, adequate freezer space, and a comparable star rating to the Sokoine Hospital.

The process of preparing these sites for the trial was comprehensive and time-consuming. It involved rigorous assessments to ensure that the facilities met the necessary standards for clinical research. Monitoring was conducted biweekly using a hybrid model that combined remote oversight with periodic on-site visits. Quality control measures included standardized data validation checks and adherence to ISO-accredited protocols for laboratory testing. Preparation activities encompassed infrastructure upgrades, staff training, and the implementation of robust quality control systems. The entire site readiness process spanned approximately 12 months, reflecting the complexity and meticulous planning needed to establish a robust and compliant trial environment.

By achieving this level of accreditation, the Lindi site now meets essential international standards and is positioned to support future clinical trials. This progress represents a significant step toward decentralizing clinical research in Tanzania and enhancing research equity by establishing sustainable capacity in historically underserved regions.

### Sponsor responsibilities in the global South

Historically, clinical trials conducted in sub-Saharan Africa have predominantly been led by research partners and sponsors from the global North. This pattern has led to a landscape where research priorities, methodologies, and funding decisions are often driven by external entities, potentially creating disparities in how research is conducted and utilized within the region. The reliance on international sponsors has sometimes resulted in a lack of local ownership and capacity-building opportunities within the host countries.

In this context, sponsorship refers to the regulatory and operational oversight role as defined by ICH-GCP guidelines, distinct from funding. While the German Federal Ministry of Education and Research (BMBF) provided financial support, NIMR assumed sponsor responsibilities including: [list specific responsibilities]. This represents a shift from traditional models where external institutions fulfill both funding and sponsor roles.

However, the TAKeOff Consortium has pioneered a transformative approach by empowering Southern partners to take on the role of sponsor. This shift has been particularly significant in the context of the filarial clinical trial, where NIMR in Tanzania assumed the role of sponsor for the study. This development marked a critical departure from traditional practices and represented a milestone in the evolution of research sponsorship in the region.

Assuming the sponsor role, NIMR was required to demonstrate significant administrative and operational capabilities. It involved overseeing the study’s design, implementation, and compliance with both international and local regulations. This responsibility included ensuring that all aspects of the trial adhered to GCP standards, managing budgets, coordinating with research sites, and maintaining high standards of ethical conduct throughout the study.

The success of NIMR in this role underscores the Institute’s growing expertise and capacity in managing complex clinical trials. This trial underscores the importance of empowering local research institutions to lead clinical trials, fostering sustainability, and reducing dependence on external actors in LMICs. It highlights the potential for local institutions in the Global South to not only participate in but also lead clinical research efforts. By taking on the sponsor role, NIMR has not only contributed to the advancement of medical research in Tanzania but has also paved the way for other Southern institutions to assume similar responsibilities in future trials.

This shift towards local sponsorship has several key benefits. It fosters a greater sense of ownership and accountability among local research teams, enhances the sustainability of research initiatives, and builds long-term research infrastructure within the region. Furthermore, it supports the development of local expertise, which is essential for addressing health challenges specific to the region.

The successful execution of the sponsor role by NIMR serves as a valuable model for other institutions in the Global South. It demonstrates that local entities can effectively manage and lead clinical trials, provided they have the appropriate support and resources. The importance of building clinical trial capacity in Africa is increasingly recognized as critical for addressing health challenges and improving pandemic preparedness. As highlighted by Ndembi in 2024 [13], less than 3% of global clinical trials are conducted in Africa, largely due to insufficient infrastructure, limited funding, and regulatory barriers. Initiatives such as the Consortium for COVID-19 Vaccine Clinical Trials (CONCVACT) and the Pan-African Clinical Trials Registry (PACTR) have demonstrated the potential of regional collaboration and capacity-building efforts in overcoming these challenges [13]. Similarly, the LeDoxy Trial underscores the necessity of strengthening local research institutions, promoting ownership, and fostering sustainability in clinical research. These efforts not only enhance health systems but also position African institutions to make effective contributions to global health research and innovation. This approach strengthens the local research ecosystem, fosters regional self-reliance and contributes to more equitable and impactful research outcomes.

Ultimately, the TAKeOff Consortium’s decision to empower Southern partners as sponsors represents a significant advancement in clinical research. It exemplifies how local institutions can successfully undertake leadership roles in research, driving forward the development of medical knowledge and improving health outcomes within their own regions while advancing global equity.

### Sustainability and long-term capacity building

Recognizing the importance of sustainable research infrastructure beyond the trial period, several mechanisms were established to ensure long-term maintenance and continued research capacity. A formal memorandum of understanding was developed with the regional health authorities, transferring ownership of key equipment (centrifuge, freezers, and temperature monitoring devices) to the health facilities upon completion of the trial. This arrangement ensured that the upgraded infrastructure would continue to benefit the local healthcare system and support future research initiatives.

To address ongoing maintenance challenges, local technical staff were trained in equipment operation and basic maintenance procedures during the trial period. Additionally, service contracts were established with regional equipment suppliers to provide continued technical support for critical equipment including the -80°C freezers and laboratory instruments. The enhanced internet connectivity installed during the trial was maintained through partnerships with local telecommunications providers, enabling continued data management capabilities in subsequent research activities.

The laboratory upgrades, which contributed to the facility’s advancement from a 2-star to 4-star rating, were designed with sustainability in mind. The accreditation processes and quality management systems implemented during the trial were integrated into the facility’s standard operating procedures, ensuring continued compliance with international standards. Regular follow-up assessments were scheduled to monitor the maintenance of these standards and provide additional support as needed. However, sustained adherence to quality standards in resource-limited settings requires ongoing financial support, technical mentorship, and institutional commitment that extends well beyond individual trial periods.

These sustainability measures enabled the Lindi research site to transition from LeDoxy trial implementation to early post-trial interventional research activity. In 2025, the site commenced a TAKeOFF Consortium–led interventional study evaluating doxycycline and moxidectin plus albendazole under a test-and-treat strategy for lymphatic filariasis elimination in Tanzania. The study, led by NIMR in collaboration with the Ministry of Health, was ongoing at the time of manuscript submission and builds directly on laboratory, clinical, and human resource capacities established during the LeDoxy Trial.

### Impact on laboratory standards

A noteworthy achievement was the substantial improvement in the laboratory’s rating. The laboratory at Sokoine Regional Referral Hospital, previously rated 2 stars, achieved a remarkable 4-star rating within 12 months following targeted infrastructure upgrades and comprehensive staff training. Additionally, the laboratory initiated the process of obtaining ISO 15189:2012 accreditation from the Southern African Development Community Accreditation Services (SADCAS), further demonstrating its commitment to excellence in clinical research. These improvements not only enhanced the laboratory’s operational quality but also contributed to the trial’s overall success, which included a 92% participant retention rate resulting from tailored follow-up strategies and robust community engagement efforts.

These infrastructure improvements were instrumental in establishing a robust research platform. They not only ensured compliance with the required standards but also laid a strong foundation for future research endeavors. By enhancing both physical infrastructure and operational capabilities, the study sites were better equipped to conduct high-quality research, contributing to the overall success of the trial.

### Human resource development and training

**Fig 2:**
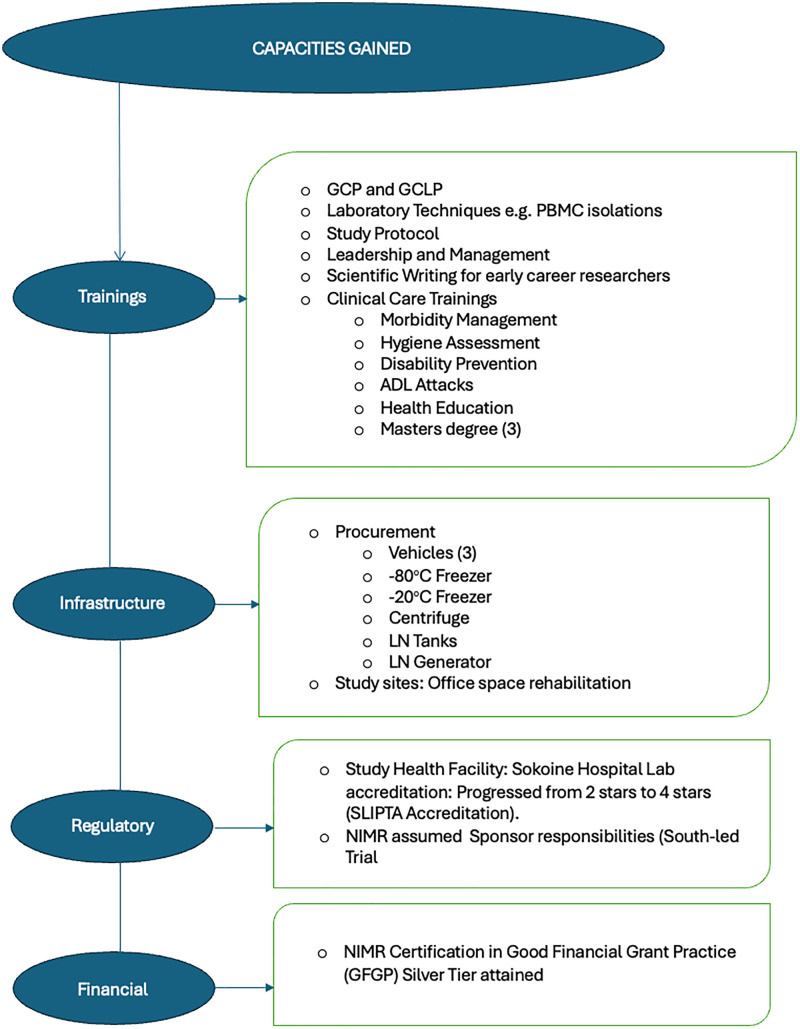
Capacity building framework for the LeDoxy Trial.

To ensure the successful implementation of the trial, a comprehensive and integrated human resource development and training program was implemented for healthcare professionals and research staff involved in the LeDoxy Trial. The program was designed to equip personnel with the clinical, research, and managerial competencies required to deliver high-quality patient care while ensuring rigorous adherence to clinical trial standards. Fig 2 illustrates the Capacity Building Framework implemented during the trial, highlighting the interconnected training components and their alignment with the trial’s objectives.

The LeDoxy Trial placed strong emphasis on capacity building through targeted training of healthcare professionals and research personnel across multiple competency domains. One key focus area was the clinical management of lymphatic filariasis (LF), where participants received specialized instruction on morbidity management, hygiene assessment, acute dermatolymphangioadenitis (ADL) attack prevention, disability mitigation, and patient-centered hygiene education. This component aimed to strengthen both clinical knowledge and practical skills, enabling frontline providers to deliver consistent evidence-based care for LF-related health outcomes in the community.

In parallel, a structured protocol and clinical research training programme was delivered to prepare staff for participation in a regulated clinical trial environment. The program was delivered by both local and international facilitators, covering essential aspects of clinical research conduct, including study procedures, data collection, and ethical considerations. Given that many participants were new to formal clinical research, special attention was given to the principles of GCP and Good Laboratory Practice (GLP). Informed consent procedures were addressed in detail, ensuring that all team members clearly understood their responsibilities to uphold ethical standards and safeguard participant rights throughout the trial.

As part of the morbidity management training, hygiene training kits were distributed to patients with lymphoedema to support the practical application of hygiene-based care. Hygiene training kits were provided to patients with lymphedema, containing essential items such as buckets, basins, medicated soap, towels, soap dishes, antifungal creams, and antibiotic creams. These kits were intended to facilitate daily hygiene practices essential for managing lymphedema and preventing complications.

Overall, a total of seventy-three healthcare workers from the study implementation areas participated in the training program alongside twenty staff members from NIMR in its role as study sponsor. This inclusive approach ensured that both site-level implementers and sponsor representatives were equipped with a shared understanding of clinical care standards, research procedures and trial governance requirements.

The integrated training programme was instrumental in developing a competent and motivated team capable of supporting both clinical service delivery and high-quality research implementation. Beyond enabling the smooth execution of the LeDoxy Trial, these investments contributed to strengthening local human resource capacity and laid the foundation for sustainable research and clinical practice improvements within the participating regions.

### Stakeholder engagement

Prior to initiating the trial, strategic efforts were undertaken to engage regional and district health authorities in Lindi, Tanzania. Recognizing the importance of local buy-in, the LeDoxy trial team implemented a comprehensive stakeholder and community engagement strategy to ensure acceptance, address concerns, and foster trust and collaboration throughout the study period.

The engagement process began with consultative meetings involving regional and district health leadership, which helped contextualize the trial within local health priorities and established critical partnerships. This was followed by a social mobilization strategy designed to inform communities about the trial’s purpose, procedures, and ethical safeguards. Multiple communication channels were utilized including community meetings, printed materials, and local radio segments to ensure broad awareness and understanding.

A key component of the strategy was the integration of a rumor monitoring and response mechanism within the existing health system. This enabled the trial team to identify emerging concerns in real-time and take appropriate corrective action. CHWs, who played a central role in this process, engaged directly with households and local groups to provide accurate information and respond to questions. These interactions also helped surface common misconceptions, such as confusion around eligibility criteria or trial procedures, which were then addressed through targeted follow-up and small-group dialogue sessions.

Additional stakeholder meetings with caregivers, elected leaders, healthcare providers, and opinion leaders served as platforms for transparent communication and feedback. Across these engagements, particular emphasis was placed on the scientific, ethical, and medical standards guiding the trial, reinforcing community confidence in the research process.

Stakeholder engagement remained a continuous process throughout the trial. Annual stakeholder meetings provided regular updates and incorporated feedback from key partners. Participation in the National NTDs Dissemination Forum, jointly organized by the NIMR and the National NTD Program, enabled broader dialogue with policymakers, researchers, and community representatives.

**Fig 3:**
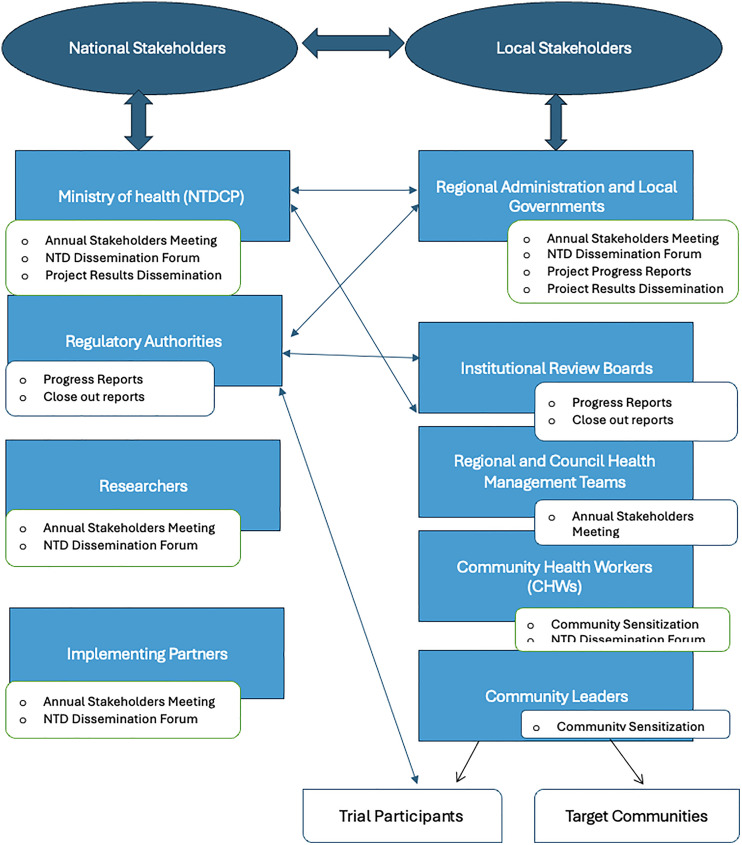
Stakeholder and community engagement activities and communication channels.

This multi-level engagement approach ensured the successful integration of the trial into the local health system and contributed to a positive perception of the research activities. By fostering open communication, prioritizing responsiveness, and embedding trusted community structures into the engagement process, the LeDoxy trial secured essential community support and helped strengthen local readiness for future research efforts. Fig 3 outlines the Stakeholder and Community Engagement Activities and Communication Channels.

### Recruiting and consenting participants

Informed consent is a fundamental process in clinical trials, ensuring that potential participants are fully aware of the study’s nature, objectives, procedures, potential risks and benefits, and any alternative treatment options available [14,15]. The effective recruitment and retention of study participants are critical to the success of clinical trials, as they can significantly impact the timeline and costs of the study [16,17]. To address these challenges, the LeDoxy trial employed a strategic approach to participant recruitment and retention.

To streamline and expedite participant enrollment, the study team developed a targeted, participant-focused recruitment plan. This strategy aimed to effectively engage potential participants while proactively addressing barriers to participation, including misconceptions and logistical constraints. Central to this effort was the involvement of CHWs, who played a pivotal role throughout the recruitment process. Their deep familiarity with local residents and established relationships within the community positioned them as trusted intermediaries, enhancing the credibility and acceptability of the trial. The critical role of community-directed distributors in NTD control has been well documented across sub-Saharan Africa, with millions of community health workers contributing to the successful delivery of interventions for neglected tropical diseases including lymphatic filariasis over more than two decades [18].

Participants were more receptive to being approached by CHWs, who were viewed as reliable and familiar figures. Working closely with the site clinical team, CHWs engaged directly with community members to explain the study’s purpose, procedures, and potential benefits. This collaborative outreach not only supported efficient participant recruitment but also contributed to building community trust and reinforcing transparency throughout the trial process.

During the informed consent process, potential participants were provided with comprehensive information about the study. This included clear explanations of the study’s objectives, procedures, potential risks and benefits, as well as available alternative treatments. The process was designed to ensure that participants could make fully informed decisions regarding their involvement. Open and ongoing communication between the research team and participants was a key component of the consent strategy. The research team prioritized accessibility and transparency, ensuring that participants could consult with study staff and receive support whenever they had questions or concerns. This approach helped reinforce participants’ confidence and trust in the research process, upholding ethical standards throughout the study.

### Participant retention and follow-up

To minimize dropout rates and loss to follow-up, the study team implemented a structured retention plan that emphasized consistent communication and participant support throughout the trial. Regular contact with participants was maintained to ensure they remained informed, engaged, and connected to the research team. CHWs played a central role in supporting retention efforts. Beyond their initial involvement in recruitment, CHWs maintained regular engagement with participants by offering continued support, including hygiene education and the distribution of antimicrobial and antifungal creams for the management of lymphedema. Their ongoing presence helped foster trust and promote adherence to the study protocol. An important operational insight gained during the study was the need to consider the local economic and occupational context. Many community members were involved in seasonal farming and fishing activities, which significantly influenced their availability. By understanding and adapting to these seasonal patterns, the study team was able to optimize the timing of follow-up visits and reduce the risk of missed appointments or participant attrition.

By leveraging the expertise and relationships of CHWs, maintaining open communication, and tailoring recruitment and retention strategies to the local context, the LeDoxy trial effectively managed participant recruitment and retention. This approach not only facilitated the successful execution of the study but also built strong community support and trust, contributing to the overall success of the trial.

### Impact of the COVID-19 pandemic on trial implementation

The onset of the COVID-19 pandemic during the recruitment phase of the LeDoxy trial introduced significant challenges to the health sector, profoundly disrupting research activities and impacting every aspect of trial implementation, from regulatory approvals to participant engagement.

The pandemic coincided with the trial’s early stages, overwhelming regulatory authorities with a surge of COVID-19-related clinical trial applications, which delayed approvals for ongoing trials, such as LeDoxy. The study team mitigated this through sustained dialogue with regulatory and ethics bodies, adapting to revised guidelines and public health protocols. Heightened concerns about COVID-19 transmission among participants and staff prompted the implementation of stringent infection control measures, including minimizing in-person interactions, mandating personal protective equipment, and implementing safety-focused procedures. Travel restrictions further complicated planned monitoring, leading to the adoption of remote monitoring via video conferencing tools (e.g., iPads, laptops, smartphones), enabling real-time oversight with an external monitor in Kenya. Global supply chain disruptions also delayed essential material deliveries, necessitating flexible timeline adjustments and innovative logistics planning.

Despite these obstacles, the LeDoxy trial demonstrated resilience by integrating pandemic response into its operations. In alignment with Tanzanian government directives, the team contributed to the national COVID-19 effort by developing and distributing tailored information, education, and communication (IEC) materials, enhancing community awareness and trust in both research and public health systems. This dual focus on trial continuity and public health support underscored the importance of adaptability, leveraging existing engagement infrastructure (e.g., community health workers) to maintain a 92% retention rate. The experience strengthened the trial’s protocols, fostering innovation, regulatory collaboration, and technological integration, and provided valuable lessons for conducting resilient clinical trials in low-resource settings during future public health crises.

## Discussion

The LeDoxy Trial represents a pioneering effort to establish a robust clinical research platform in Tanzania’s resource-limited Lindi region, demonstrating the feasibility of conducting high-quality trials in underserved settings. While its clinical outcomes, evaluating doxycycline’s efficacy for filarial lymphedema, are detailed in Ngenya et al. [10], this study illuminates the strategic approaches that enabled its success, including laboratory upgrades from a 2-star to a 4-star rating, a 92% participant retention rate, and the training of 93 healthcare professionals. By leveraging local sponsorship through the NIMR and fostering community trust through CHWs, the trial not only addressed a critical gap in lymphatic filariasis research but also offers transferable lessons for advancing clinical research capacity across other NTDs and LMIC contexts.

The clinical research landscape in Tanzania and other LMICs faces distinct challenges and opportunities. While pre-service training in medical and allied health professions in Tanzania traditionally emphasizes routine health services, there has been a noticeable shift towards fostering a research culture. Despite recent efforts to integrate research and publication into professional development through Continuing Professional Development (CPD) points, the overall engagement in research remains limited. This paper highlights both the progress and ongoing challenges in advancing a research-oriented medical community in Tanzania.

Medical training in Tanzania has historically been focused on clinical service and education rather than research. While there is a growing trend towards research involvement among clinicians, the numbers remain relatively low. The linkage of professional registrations with CPD points from research activities represents a positive step toward encouraging research engagement. However, retaining trained research staff remains a significant challenge [19,20]. Research positions, often temporary, may not offer the same stability or incentives as permanent roles, leading to high turnover rates. To address this, offering incentives such as professional development opportunities, short courses, and advanced degrees can be crucial for retaining skilled researchers.

Cultural and regulatory environments have a significant influence on the success of clinical trials. Differences in cultural perceptions and practices can present barriers [21], especially when dealing with diseases that carry social stigma or are associated with local beliefs, such as curses. Understanding and addressing these cultural nuances is critical for the effective implementation of trials. One effective practice identified in this study was the appointment of a dedicated internal regulatory officer. This role is pivotal in minimizing application delays, managing deviations, and supporting monitoring functions, ensuring smoother regulatory processes and compliance.

Conducting trials in rural areas presents unique logistical challenges. Seasonal factors, such as the rainy season, can impact transportation and access to participants, while religious observances, such as Ramadan, can complicate adherence to strict follow-up schedules. To mitigate these issues, study protocols should incorporate flexible follow-up arrangements to address potential concerns regarding ethics, safety, and data bias. This flexibility helps ensure that participant engagement and data integrity are maintained despite logistical and cultural challenges [22].

The involvement of CHWs was a crucial factor in the successful recruitment and retention of participants [23]. CHWs, who are well-integrated into their communities, played a vital role in identifying potential participants, providing support, and facilitating communication between the research team and the community. Their established presence and ongoing training were instrumental in overcoming local challenges and ensuring the smooth operation of trials. This approach aligns with decades of successful community-directed treatment strategies for NTD control across sub-Saharan Africa, where community-directed distributors have proven to be essential “foot soldiers” in achieving high coverage and sustained participation in mass drug administration programs [24]. Their established presence and ongoing training were instrumental in overcoming local challenges and ensuring the smooth operation of trials.

The LeDoxy Trial, conducted in Tanzania, provides a robust framework for clinical research in LMICs, with lessons that extend well beyond the management of filarial lymphedema. The trial’s success in navigating a complex research landscape characterized by limited infrastructure, cultural nuances, and logistical constraints offers strategies that are highly adaptable to other disease areas, particularly those prevalent in resource-limited settings.

One key lesson is the trial’s emphasis on community engagement, which leveraged CHWs to facilitate participant recruitment and retention. This approach can be applied to clinical trials for other NTDs, such as schistosomiasis or soil-transmitted helminths, where community trust is essential for successful implementation. For example, in malaria vaccine trials, community engagement has been important in overcoming mistrust and achieving high participation rates, as observed in studies conducted in sub-Saharan Africa [25]. Similarly, in HIV/AIDS research, where stigma and cultural sensitivities often hinder participation, the LeDoxy Trial’s strategy of involving local leaders and tailoring communication to local contexts can enhance trial adherence and outcomes.

The trial’s flexible protocol design, which accommodated seasonal and religious factors affecting participant availability, is another transferable strategy. This flexibility is particularly relevant for diseases requiring long-term follow-up, such as tuberculosis, where consistent patient engagement in rural or resource-limited settings can be challenging due to geographical and infrastructural barriers. By adapting protocols to local conditions, the LeDoxy Trial offers a model for ensuring trial feasibility in diverse LMIC settings.

Furthermore, the trial’s focus on capacity building, including training local staff and empowering NIMR as the trial sponsor, underscores the importance of investing in local research infrastructure. This approach can be replicated in trials for non-communicable diseases such as diabetes or hypertension, where sustainable research capacity is needed to address growing health burdens in LMICs. For instance, trials for chronic conditions often require ongoing monitoring and local expertise, which can benefit from the training and infrastructure development strategies employed in the LeDoxy Trial.

To highlight the unique contributions of the LeDoxy Trial, it is useful to compare it with other clinical trials conducted in LMICs, particularly those addressing NTDs or similar health challenges. An example is the NIDIAG consortium, a European research network that researchers to improve the quality of neglected infectious disease care at primary health care level in resource-poor settings, conducted diagnostic trials for febrile syndromes and persistent digestive disorders across multiple LMICs, including Cambodia, the Democratic Republic of Congo, Indonesia, Ivory Coast, Mali, Nepal, and Sudan [25]. These trials faced challenges similar to those encountered in the LeDoxy Trial, including geographical inaccessibility, limited electricity and internet connectivity, shortages of qualified research staff, and the vulnerability of the communities. To address these, the NIDIAG trials implemented solutions such as pre-study assessments to tailor training and infrastructure needs, negotiated budget flexibility with funders, and developed risk-adapted monitoring strategies.

The LeDoxy Trial is not the first to establish clinical research capacity in remote African settings. Notably, the moxidectin Phase 3 trial (2009–2012) created three new clinical research centers one in Liberia and two in the Democratic Republic of the Congo in post-conflict areas with no prior clinical research infrastructure, while also strengthening an existing center in Ghana [24,26]. These centers were established specifically to conduct trials in remote onchocerciasis-endemic communities and have since supported additional research for NTD control. Similarly, research platforms established through initiatives such as the NIDIAG consortium have addressed comparable infrastructure and capacity challenges across multiple LMICs [25]. However, the LeDoxy Trial makes a distinct contribution by providing comprehensive, integrated documentation of the entire infrastructure development process from laboratory accreditation and regulatory navigation to community engagement and sustainability planning within a single, detailed account focused on lymphatic filariasis morbidity management in Tanzania.

The LeDoxy Trial employed comparable strategies, including utilizing CHWs to address recruitment challenges and establishing comprehensive training programs for local staff. However, it distinguishes itself through its specific focus on filarial lymphedema, a chronic morbidity that is often overshadowed by mass drug administration (MDA) programs aimed at interrupting transmission. By investigating doxycycline as a treatment option, the LeDoxy Trial addresses a critical gap in managing the long-term complications of lymphatic filariasis, which affects millions globally [27–30].

Another unique contribution is the LeDoxy Trial’s emphasis on local leadership, with NIMR taking on the sponsor role. This approach aligns with broader calls to strengthen research capacity in LMICs. By empowering Tanzanian researchers and institutions, the LeDoxy Trial contributes to closing this gap and promoting equitable participation in global health research [31].

The experiences and best practices gleaned from this trial offer valuable insights that can be adapted to other clinical research contexts, including those beyond filarial studies. Effective strategies for recruitment, community engagement, and protocol flexibility, as well as addressing logistical and cultural barriers, can be applied to a wide range of clinical trials in LMICs.

## Limitations

This analysis has limitations that should be considered when interpreting the findings. The work was not designed as a comparative or controlled study, and the observations presented cannot be benchmarked against alternative trial sites or implementation models. Consequently, the findings are descriptive and observational in nature rather than inferential. The analysis is also based on experiences from a limited number of study sites within one region of Tanzania, which may constrain the extent to which the findings are generalizable to other remote or underserved settings with different contextual characteristics.

The duration of post-trial follow-up was limited, restricting the ability to assess the long-term sustainability of staff retention, continued use of strengthened infrastructure, and integration of newly developed research capacities into routine health services. In addition, because the analysis relies on retrospective synthesis of routine trial documentation and implementation experiences, there is potential for observer and interpretive bias, despite efforts to triangulate findings across multiple sources and perspectives. Finally, no formal cost-effectiveness analysis was undertaken to compare the establishment and operation of clinical trial sites in remote versus more urbanized settings, limiting conclusions regarding relative efficiency or resource implications.

Despite these limitations, the strength of this work lies in its detailed documentation of real-world implementation experiences. Rather than aiming for statistical inference, the findings are intended to support transferability by offering practical insights that may inform the planning and execution of clinical trials in similar resource-constrained contexts.

## Conclusion

The LeDoxy Trial illustrates that it is feasible to establish and operationalize high-quality clinical research platforms in remote, resource-limited settings through deliberate investments in infrastructure, human resource development, and community engagement. By documenting the processes, challenges, and solutions encountered during trial implementation, this paper provides practical insights into how clinical research capacity can be built in settings with limited prior research exposure. The experience from Lindi underscores the importance of locally led sponsorship, context-responsive training, and sustained engagement with health systems and communities as foundational elements for successful trial implementation. While grounded in a specific context, the approaches described are transferable and may inform the planning and execution of interventional clinical trials in other underserved settings.

Sustaining research capacity beyond individual trial periods remains a critical priority. that requires longitudinal evaluation. While early post-trial experience suggests promise, the long-term retention of trained personnel in remote settings, maintenance of upgraded infrastructure, and full integration of research functions into routine health services remain to be systematically documented. Continued investment, ongoing mentorship, and sustained institutional commitment will be essential to ensure that gains achieved through individual trials translate into lasting improvements in research readiness and health system strengthening. Future research should include formal sustainability assessments at 5- and 10-year intervals post-trial to provide robust evidence on the durability of capacity-building investments.
