# Correction: Establishing a filarial clinical research platform in a resource-limited setting: Lessons and experiences from Tanzania

**DOI:** 10.1371/journal.pntd.0014236

**Published:** 2026-04-20

**Authors:** Ndekya M. Oriyo, Michael A. Munga, Abdallah Ngenya, John Ogondiek, Wilfred Mandara, Winfrida John, Jonathan Akyoo, Ruth Laizer, Mathias Kamugisha, Hanifa Kapera, Rafikieli Ngwatu, Gracia Sanga, Jacklina Mhidze, Antelmo Haule, Anja Feichtner, Haile Mposheleye, Farida A. Mwanga, Hatima A. Chinguile, Alexander J. Makalla, Peter Mabenga, Max Demetrius, Maureen M. Mosoba, Dennis Moshi, Yusuph Mgaya, Linda Batsa Debrah, Inge Kroidl, Ute Klarmann-Schulz, Samuel Wanji, Alexander Yaw Debrah, Achim Hoerauf, Akili Kalinga, Upendo Mwingira

The sixth author’s name is spelled incorrectly. The correct name is: Winfrida John. The correct citation is: Oriyo NM, Munga MA, Ngenya A, Ogondiek J, Mandara W, John W, et al. (2026) Establishing a filarial clinical research platform in a resource-limited setting: Lessons and experiences from Tanzania. PLoS Negl Trop Dis 20(2): e0013983. https://doi.org/10.1371/journal.pntd.0013983
